# Revisiting Miller’s pyramid in medical education: the gap between traditional assessment and diagnostic reasoning

**DOI:** 10.5116/ijme.5d9b.0c37

**Published:** 2019-10-25

**Authors:** Annamaria Witheridge, Gordon Ferns, Wesley Scott-Smith

**Affiliations:** 1 Division of Medical Education, Brighton and Sussex Medical School, Falmer, Brighton, UK

**Keywords:** Miller’s pyramid, medical education, traditional assessment, diagnostic reasoning

## Introduction

In 1990, George Miller outlined a new model for the assessment of clinical competency.^1^ Miller argued that the traditional assessment of medical students relied too much on testing their knowledge, and not enough on assessing how they would behave in a real-life consultation.^1^ His model, known as the ‘Miller pyramid’, was the basis for an important move away from the traditional Flexnerian medical education model that was largely dominated by theoretical knowledge-based assessments, towards examinations based on clinical performance.

Miller’s pyramid model divides the development of clinical competence into four, hierarchical processes.^1^ On the lowest level of the pyramid is ‘knowledge’, tested by written exams and traditional multiple-choice questions (MCQs).^2^ The next level stands for ‘application of knowledge’, assessed by essays, clinical problem-solving exercises and extended MCQs.^2^ The third tier of the pyramid represents ‘clinical skills competency’, assessed by standardized patient exercises, simulations and clinical exams.^2^ Finally, on top of the pyramid is ‘clinical performance’, assessed by direct observation in real clinical settings.^2^ The lower level processes account for the cognitive components of competence and involve classroom-based assessments, while the two higher tiers of the pyramid account for the behavioural components of clinical competence, which involve assessment in simulated and real clinical settings.^3^  By placing the observable behaviour at the apex of the hierarchy, Miller’s pyramid implies a preference towards the behaviourist tradition, as opposed to cognitive perspectives. Miller’s model suggested that simulated practice could provide a good approximation to how students would behave in the real-world clinical setting, and therefore firmly argued for the introduction of performance-based assessments.

In the second half of the 20^th^ century, the ‘performance-as-competence’ paradigm led to an increased use of a new form of assessment in medical training, known as Objective Structured Clinical Examinations (OSCEs). These examinations were designed to test the clinical competence of trainees in a novel way, through the observation of participants across a series of timed stations that could involve history taking, physical examination or patient management, evaluated through standardized checklists, providing a level of objectivity. Today, OSCEs are one of the most widely used assessment methods in medical education.

However, their wide adoption to assess clinical competencies does not reflect their inherent limitations. Caution should be particularly exercised when new skills are being added, as OSCEs may not be suitable for the assessment of all clinical skills^4^^,^^5^ and a fundamental problem arises when OSCEs are used for the assessment of cognitive skills, such as diagnostic reasoning.

Diagnostic reasoning refers to the cognitive processes involved in diagnosis and management in medical practice. The recent increase in awareness of the importance of this skill has stimulated medical schools to integrate diagnostic reasoning into their curricula.^6^ In the absence of a more appropriate assessment method, it has often been added to the list of competencies that OSCEs may be used to assess.^7^^,^^8^ However, this ignores the fundamental fact that according to Miller’s pyramid, OSCEs were never intended to assess cognitive skills in the first place. Miller’s pyramid clearly indicates that standardized patient exercises, such as OSCEs are designed to assess observable behaviour, rather than cognitive skills. The problem with using traditional, observation-based assessment for diagnostic reasoning is that it does not allow assessment of the process of diagnostic reasoning, only the distal outcome of this process. It can only assume the reasoning process based on the questions being asked and the diagnosis and management being formulated. However, asking the right questions or stating the right diagnosis does not necessarily mean that the reasoning process itself was correct.^9^ Without further investigating why a student is asking those particular questions, and how the information they are collecting is being interpreted in the light of their working diagnosis, it may not be possible to differentiate students with differing degrees of diagnostic reasoning investment.^9^ OSCEs do not provide an opportunity for students to overtly express their reasoning process, the discrepancy between what students think and what they say out loud can easily be misinterpreted, which renders such method unreliable. This is consistent with emerging evidence showing that OSCE performance does not necessarily align with diagnostic reasoning skills.^10^^,^^11^ A quantitative study of sixty-five fourth-year medical students has found that OSCE performance scores do not correlate with diagnostic reasoning or diagnostic accuracy scores.^10^ This study therefore concluded that traditional OSCE scores do not reflect the students’ diagnostic reasoning ability.^10^ A recent qualitative study has also found that fourth-year medical students’ diagnostic approach during OSCE-style consultations do not reflect their true diagnostic ability.^11^ Whilst it is important to acknowledge the inherent limitations of these early studies, such as their cross-sectional nature and single-centre design with small participant numbers, their findings should not be dismissed. They highlight an important discrepancy between OSCE performance and diagnostic reasoning ability, which may be due to the fundamental misalliance of using an observational assessment method to evaluate a covert, complex cognitive skill.

Diagnostic reasoning is an essential part of clinical competency, and the theoretical framework for clinical competency assessment needs to take this into account. In order to achieve this, the cognitive elements of the Miller pyramid need to be given more emphasis. While the competencies of ‘knowledge’ and ‘application of knowledge’ on the lower two tiers are essential for assessing medical knowledge in classroom settings, the cognitive element should not stop there. Due to the context dependent nature of diagnostic reasoning, it is important for learners to demonstrate their reasoning process of solving diagnostic problems in a clinical context. Therefore, the upper tiers of Miller’s pyramid need to incorporate ‘sound diagnostic reasoning’ assessed first in simulated, and then in the real-world, clinical setting.  Revising Miller’s pyramid by placing greater emphasis on the cognitive elements of competency, could be the first step towards developing an assessment strategy that values the importance of diagnostic reasoning. Considering the importance of sound diagnostic reasoning skills for safe clinical practice, it is essential that medical graduates entering clinical practice are assessed on this skill in order to make sure they have the necessary skills to practice safely in their new role as junior doctors. Therefore, it is important to put in place adequate assessment methods in undergraduate medical education, which focuses on the use of diagnostic reasoning. Based on the arguments discussed here, it is important to consider how existing assessment methods, such as OSCEs could be modified to better suit the covert nature of diagnostic reasoning skills. Combining think-aloud protocols with real or simulated practice may overcome the limitations of current practices. Instructing the students to verbalize their reasoning process pre- and post-consultation may increase their engagement in active diagnostic reasoning during the diagnostic task and thereby facilitate the development of their diagnostic skills.

### Conflict of Interest

The authors declare that they have no conflict of interest.
